# Environmental Reconstruction of Tuyoq in the Fifth Century and Its Bearing on Buddhism in Turpan, Xinjiang, China

**DOI:** 10.1371/journal.pone.0086363

**Published:** 2014-01-27

**Authors:** Ye-Na Tang, Xiao Li, Yi-Feng Yao, David Kay Ferguson, Cheng-Sen Li

**Affiliations:** 1 State Key Laboratory of Systematic and Evolutionary Botany, Institute of Botany, Chinese Academy of Sciences, Beijing, China; 2 School of Chinese Classics, Renmin University of China, Beijing, China; 3 Department of Paleontology, University of Vienna, Vienna, Austria; University of Oxford, United Kingdom

## Abstract

The Thousand Buddha Grottoes of Tuyoq, Turpan, Xinjiang, China were once a famous Buddhist temple along the ancient Silk Road which was first constructed in the Fifth Century (A.D.). Although archaeological researches about the Grottoes have been undertaken for over a century, the ancient environment has remained enigmatic. Based on seven clay samples from the Grottoes’ adobes, pollen and leaf epidermis were analyzed to decipher the vegetation and climate of Fifth Century Turpan, and the environmental landscape was reconstructed in three dimensions. The results suggest that temperate steppe vegetation dominated the Tuyoq region under a warmer and wetter environment with more moderate seasonality than today, as the ancient mean annual temperature was 15.3°C, the mean annual precipitation was approximately 1000 mm and the temperature difference between coldest and warmest months was 24°C using Co-existence Approach. Taken in the context of wheat and grape cultivation as shown by pollen of *Vitis* and leaf epidermis of *Triticum*, we infer that the Tuyoq region was an oasis with booming Buddhism in the Fifth Century, which was probably encouraged by a 1°C warmer temperature with an abundant water supply compared to the coeval world that experienced the 1.4 k BP cooling event.

## Introduction

**Figure 1:**
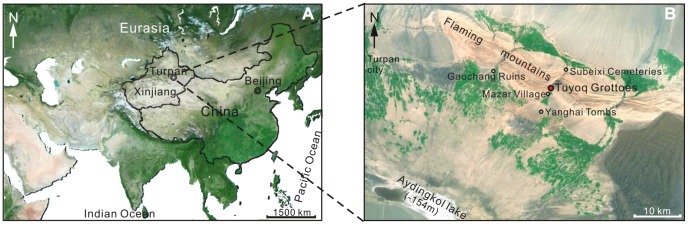
Map showing the location of Tuyoq Grottoes (revised from http://map.baidu.com/). (A) Location of Turpan in Eurasia, (B) Location of Tuyoq Grottoes in Turpan.

The Xinjiang Uygur Autonomous Region in China is located in the heart of Central Eurasia (Fig. 1), where it has been governed by an arid, temperate continental climate since the uplift of the Tibetan Plateau [1], [2]. In particular, the Turpan Basin is an extremely arid region of Xinjiang, with only 16.4 mm mean annual precipitation [3], but 3000 mm evaporation [4]. However, Turpan was once an important stop on the overland trade route ‘Silk Road’ linking China with Central Asia. The investigation of the historical vegetation and climate may provide a key to understand the people’s lifestyle in the Turpan oasis in ancient time.

Turpan has been generally characterized by an arid climate with expanding temperate desert and steppe vegetation throughout the Holocene [5], [6]. In recent years, there have been a series of archaeobotanical discoveries from the Turpan area, including Cannabis sativa [7], Capparis spinosa [8], Lithospermum officinale [9], Vitis vinifera from the Yanghai Tombs (2.5 kyrs BP) [10], *Sesamum indicum* from Boziklik Thousand Buddha Grottoes (700 yrs BP) [11], most of which were plants utilized by indigenous people for medicine, oil, spice etc. Moreover, six species of cereals including *Triticum aestivum* and Setaria italica from the Astana Cemeteries were recognized as food for local people in 3^rd^ to 9^th^ centuries [12]. Furthermore, Jiang et al. [13] and Yao et al. [14] reported the ancient Yanghai people of Turpan were living in an oasis with some swamps 2700 yrs BP ago based on an archaeological environmental study. However, there is still a shortage of research on the ancient climate and environmental reconstruction of Turpan.

The Tuyoq Grottoes of Turpan, first constructed in the Fifth Century and abandoned during the Fifteenth Century, were once a notable Buddhist pilgrimage site, even for the Tang royal family between the seventh to eighth centuries [15]. Since they were discovered by the Russian botanist E. A. Regel in the early 1870s, there have been continuous reports on the archaeological findings such as Buddhist frescoes, silk paintings, wood and pottery utensils and so on [16]–[18]. In 2010, approximately 2500 square meters of caves were excavated by a joint team of the Research Center for Frontier Archaeology of the Institute of Archaeology, Chinese Academy of Social Sciences and Academia Turfanica and Kizil Research Institute [19].

In the present study, we employ pollen and epidermis analyses to reconstruct the ancient vegetation, climate and environment of the archaeological site ‘The Thousand Buddha Grottoes of Tuyoq’ in Turpan in the Fifth Century, as well as to probe the possible historical signatures of the environment-human interaction there.

## Materials and Methods

### Ethics Statement

All necessary permits were obtained for the described field studies and were granted by the Academia Turfanica of Xinjiang Uygur Autonomous Region, China.

### Research Site

Turpan (41°12′–43°40′N, 87°16′–91°55′E) is located in an intermontane basin enclosed by high mountains in Xinjiang, NW China. The basin covers 50,140 km^2^ including a 7.8 km^2^ oasis [20], with the highest Bogeda Peak (5445 m) in the north and the second lowest lake on the planet Aydingkol Lake (−154 m) in the central basin (Fig. 1). The basin is governed by an arid, temperate continental climate, and water resources in the basin come mainly from glacial melt in the Tian and Bogeda Mountains channeled by karez irrigation systems [4]. Modern plants in the basin include camel thorn (*Alhagi sparsifolia*), sea-buckthorn (*Hippophae rhamnoides*), tamarisk (*Tamarix chinensis*), artemisia (*Artemisia ordosica*), sand ilex (*Ammopiptanthus mongolicus*) and some cultivated grape (*Vitis vinifera*), cotton (*Gossypium hirsutum*), sweet melon (*Cucumis melo*), poplar (*Populus tomentosa*), mulberry (*Morus alba*) etc. [6].

The Thousand Buddha Grottoes of Tuyoq, comprising 94 caves, are situated in the eastern and western cliff faces of Tuyoq Valley cutting through the Flaming Mountains, Shanshan County near Turpan (Fig. 1). The Grottoes lie fifteen kilometers east of the Gaochang ruins, which are regarded as the earliest example of a combined central Asiatic and Chinese style of Buddhism [15]. The Tuyoq Valley is about eight kilometers in length, with Subeixi Cemeteries in the north and Yanghai Tombs in the south. About one kilometer from the Tuyoq Grottoes, an ancient, small Uighur village called ‘Mazar Village’ is located at the southern mouth of the valley, where yellow clay was the main source of material for local building.

### Studied Materials and Methods

**Figure 2:**
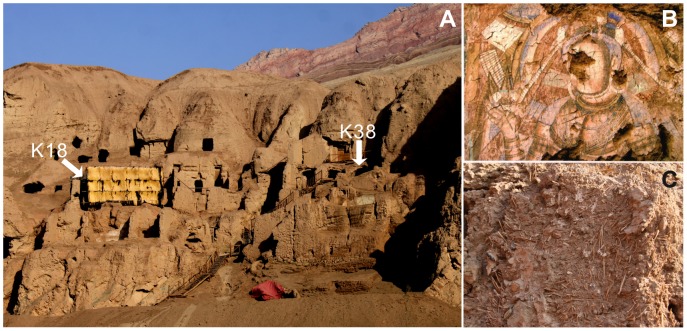
Tuyoq Grottoes sampling locality. (A) Sampling Grottoes K18 and K38, (B) Buddha fresco in the Grotto K18, (C) Picture showing plants fragments mixed in the clay adobe as strengthening materials.

Seven soil samples from intact yellow-clay adobes in the northern portion of the eastern Tuyoq Grottoes (42°51.9′N, 89°41.7′E) were selected for this study. There were diverse plant stems and leaves mixed in the clay as architectural strengthening materials (Fig. 2). Samples nos.1 to 5 were collected from Grotto K18, the central Buddhist prayer room in the eastern grottoes. Samples no.6 and no.7 collected from Grotto K38, a cave with a Buddha niche in the center (Fig. 2). According to Chinese Bodhisattva frescoes classification and archaeological research, the grottoes must have been constructed in the Fifth Century [21], [22].

Based on the technique of Heavy Liquid Separation (density: 2.0 g mL^−1^,) [23], [24], 20 g soil samples were analyzed for pollen, which were preserved in glycerin, observed under a Leica DM 2500 light microscope and photographed at 200× or 400× magnifications. The single-grain technique of Ferguson *et al.* [25] was applied for pollen observation under a Hitachi S-4800 scanning electron microscope (SEM). 3500 pollen grains from the Tuyoq Grottoes were counted in all, 500 grains from each sample. Pollen identification was based on the monographs ‘Pollen Flora of China’, ‘Pollen morphology of plants from dry and semi-arid areas in China’ and other published literature [26]–[28], and compared to the pollen of modern plants from the Chinese National Herbarium (PE) if necessary. Simultaneously, pollen of nine common Poaceae species from the modern Tuyoq region were observed under SEM to assist ancient Poaceae pollen identification. The relative abundance (RA) of a pollen taxon is calculated by the equation: RA = N/Nt (N: pollen/spore number of a taxon, Nt: total pollen/spore number in the sample).

Leaf epidermis analysis was carried out by washing plant remains from the ancient clay adobe, then preparing the epidermis in a 1∶1 mixture of 30% hydrogen peroxide (H_2_O_2_) and 99% glacial acetic acid (CH_3_COOH) at 60°C in a water bath for one or two days [29]. Finally, the epidermides were cleaned with a fine brush and washed again in distilled water until the epidermis textures were clearly visible under a stereo microscope. The epidermides were mounted on microscopical slides with glycerin for observation and measurement, followed by the same procedure as pollen observation and photography. 35 blades of leaf epidermides were analyzed from the Tuyoq Grottoes, 5 blades from each sample. The epidermides were mainly identified using the monograph ‘Micromorphological Atlas of Leaf Epidermis in Gramineae’ and other published literature [30]–[33], and compared to the epidermides of modern plants from PE if necessary. Eight structural features were employed for leaf epidermis taxonomy, including the long cells, short cells, stomatal complexes and subsidiary cell shape, prickle-hairs, macro- and micro-hairs, and silica bodies. The terminology used to describe the leaf epidermis follows Ellis (1979) [34] and Chen (1993) [30].

The ancient climate of the Tuyoq Grottoes was reconstructed following the Coexistence Approach (CA) [35], whereby the following five climatic parameters were calculated: the mean annual temperature (MAT), the mean warmest monthly temperature (MWMT), the mean coldest monthly temperature (MCMT), the temperature difference between coldest and warmest months (DT) and the mean annual precipitation (MAP). On the assumption that the climatic tolerance of an ancient taxon was similar to its nearest living relatives (NLRs), the principle of CA is to estimate the ancient climatic parameters by using the modern climatic parameters of NLRs distribution [35]. In this work, each ancient pollen taxon in the assemblage is identified to a NLR. Modern distribution of NLRs in China follows Wu & Ding (1999) [36], and the climatic tolerances of NLRs were obtained from the modern meteorological data (1951–1980) recorded at various meteorological stations in China [3], [37]–[41]. The 3D reconstruction of the Tuyoq landscape in the Fifth Century was completed using software of ‘3D Studio Max’ and ‘Adobe Photoshop’.

## Results

### Pollen Analyses

**Figure 3:**
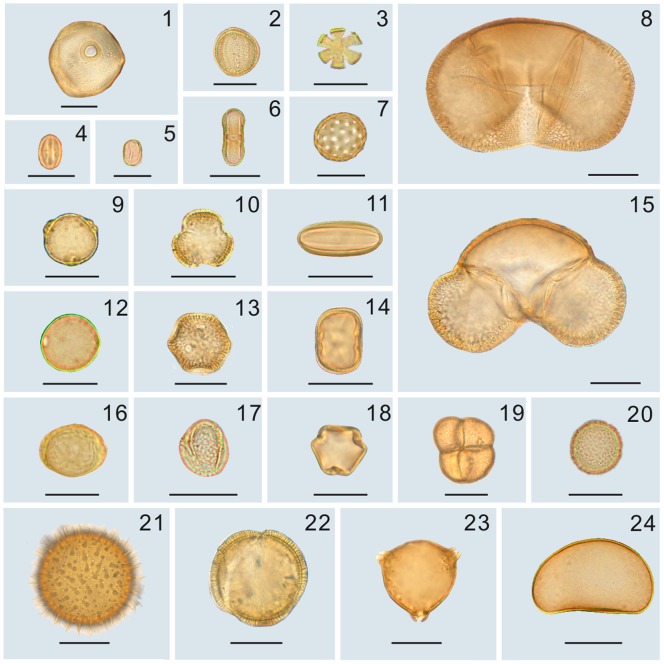
Main palynomorphs of Tuyoq Grottoes. 1. Poaceae 2. Ranunculaceae 3. Lamiaceae 4. *Castanea* 5. *Alhagi* 6. Apiaceae 7. Chenopodiaceae 8. *Picea* 9. *Betula* 10. *Artemisia* 11. *Ephedra* 12. Moraceae 13. Caryophyllaceae 14. Fabaceae 15. *Pinus* 16. *Ulmus* 17. *Salix* 18. *Vitis* 19. *Typha* 20. Potamogetonaceae 21. Malvaceae 22. Convolvulaceae 23. Elaeagnaceae 24. Polypodiaceae; Scale bar = 20 µm.

**Figure 4:**
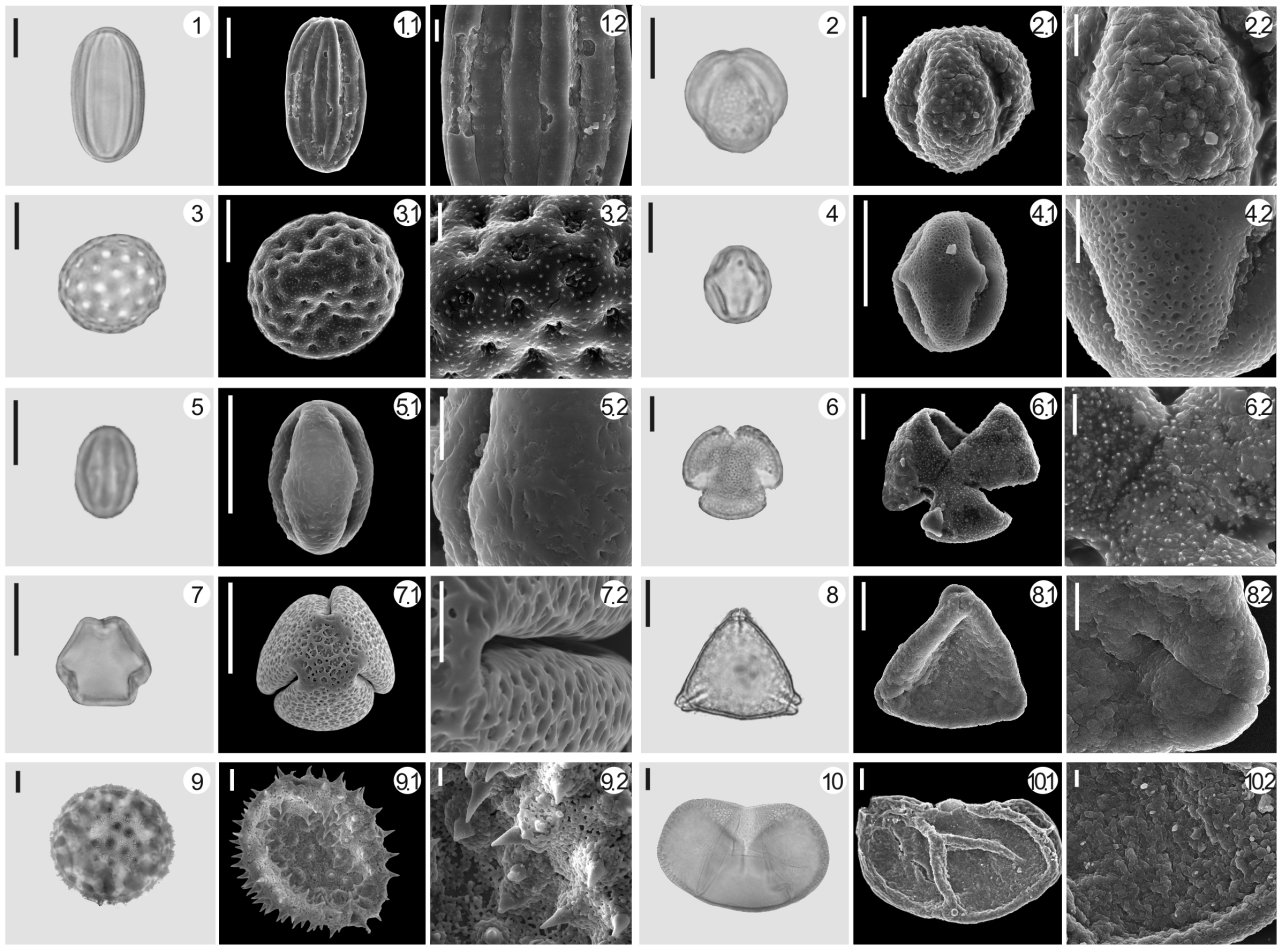
A selection of palynomorphs from Tuyoq Grottoes. 1. *Ephedra* 2. *Artemisia* 3. Chenopodiaceae 4. *Alhagi* 5. *Castanea* 6. Ranunculaceae 7. *Vitis* 8. Elaeagnaceae 9. Malvaceae 10. *Picea*; Scale bar in light microscope (LM) and scanning electron microscopic (SEM) overview 10 µm, in SEM close-up 2.5 µm.

44 palynomorphs were extracted from the samples of Tuyoq Grottoes, consisting of 38 angiosperms (85.45%), 3 gymnosperms (13.83%), 1 pteridophyte (0.43%) and 2 unknowns (0.29%) (Table 1, Figs. 3–4). The angiosperms were assigned to 31 families and the gymnosperms to 2 families.

**Table 1:** The palynomorph relative abundance (RA) of Tuyoq Grottoes.

Palynomorph			RA
Pteridophytes	Polypodiaceae		0.43%
Gymnosperms	Ephedraceae	*Ephedra*	10.69%
	Pinaceae	*Pinus*	1.89%
		*Picea*	1.26%
Angiosperms	Poaceae	*Phragmites australis*	4.00%
		other Poaceae	26.11%
	Asteraceae	*Artemisia*	19.83%
		other Asteraceae	3.03%
	Chenopodiaceae		10.71%
	Fabaceae	*Alhagi*	3.83%
		*Medicago*	0.29%
		other Fabaceae	4.71%
	Moraceae		1.80%
	Ranunculaceae		1.54%
	Ulmaceae	*Ulmus*	1.29%
	Typhaceae	*Typha*	0.97%
	Alismataceae	*Alisma*	0.91%
	Apiaceae		0.71%
	Polygonaceae	*Polygonum*	0.66%
	Salicaceae	*Salix*	0.66%
	Caryophyllaceae		0.46%
	Lamiaceae		0.40%
	Potamogetonaceae		0.37%
	Rosaceae		0.34%
	Boraginaceae		0.31%
	Elaeagnaceae		0.31%
	Malvaceae		0.29%
	Rutaceae		0.29%
	Betulaceae	*Betula*	0.20%
		*Corylus*	0.14%
	Convolvulaceae		0.20%
	Liliaceae		0.20%
	Euphorbiaceae		0.17%
	Vitaceae	*Vitis*	0.17%
	Plantaginaceae		0.09%
	Fagaceae	*Castanea*	0.14%
		*Quercus*	0.09%
		other Fagaceae	0.06%
	Rubiaceae		0.09%
	Brassicaceae		0.03%
	Celastraceae		0.03%
	Juglandaceae		0.06%
Unknown-1			0.09%
Unknown-2			0.20%
**Total**	44		100%

**Figure 5:**
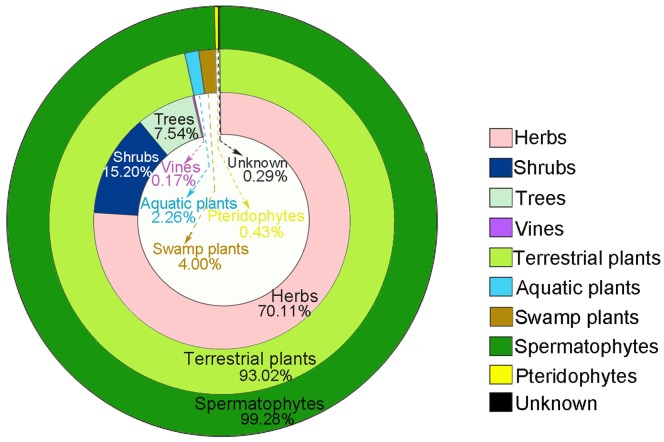
Relative abundance (%) of different plant categories of palynomorphs from Tuyoq Grottoes.

In the spermatophytes, terrestrial plants comprised 93.02%, of which 70.11% were herbs (Poaceae 26.11% , *Artemisia* 19.83%, Chenopodiaceae 10.71%), 15.20% shrubs (*Ephedra* 10.69%, *Alhagi* 3.83%, Rosaceae 0.34%), 7.54% trees (*Pinus* 1.89%, Moraceae 1.80%, *Picea* 1.26%), 0.17% vines (*Vitis*), swamp plants (*Phragmites australis*) comprised 4.00%, aquatic plants only comprised 2.26% (*Typha* 0.97%, *Alisma* 0.91%, Potamogetonaceae 0.37%) (Fig. 5).

### Epidermis Analyses

**Figure 6:**
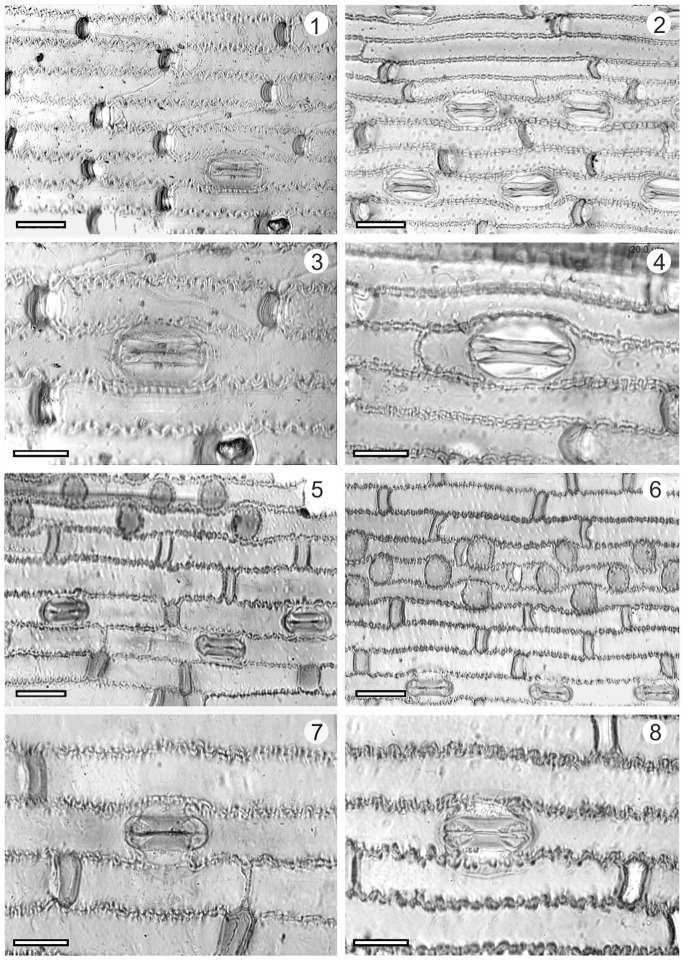
Comparison of epidermides from the Tuyoq Grottoes and modern plants. 1,3: Unearthed lower leaf epidermis of wheat (*Triticum* sp.) from Tuyoq Grottoes, 2,4: Lower leaf epidermis of modern wheat (*Triticum aestivum*), 5,7: Unearthed lower leaf epidermis of *Agropyron mongolicum* from Tuyoq Grottoes, 6,8: Lower leaf epidermis of modern *Agropyron mongolicum*; Scale bar in1,2,5,6 = 30 µm; in 3,4,7,8 = 20 µm.

**Figure 7:**
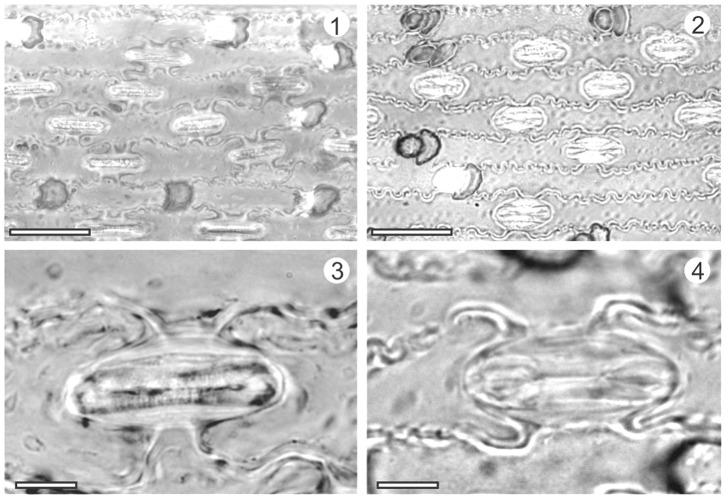
Comparison of epidermides from the Tuyoq Grottoes and modern plants. 1,3: Unearthed lower leaf epidermis of reed (*Phragmites* sp.) from Tuyoq Grottoes, 2,4: Lower leaf epidermis of modern reed (*Phragmites australis*); Scale bar in 1,2 = 20 µm; in 3,4 = 5 µm.

The leaf epidermis of *Triticum* sp. (wheat), *Agropyron mongolicum* and *Phragmites* sp. (reed) were identified from the samples of Tuyoq Grottoes (Table 2, Figs. 6–7).

**Table 2:** Plant taxa of leaf epidermis with quantitative distribution in the samples.

Plant taxa	Sample no. 1	no.2	no.3	no.4	no.5	no.6	no. 7	Total
*Phragmites* sp.	0		2	0	1	3	1	7
*Triticum* sp.	4	2	3	3	3	2	3	20
*Agropyron mongolicum*	0	2						2
Unknown	1	1		2	1		1	6
**Total**	5	5	5	5	5	5	5	35

1. *Triticum* sp. (Family: Poaceae, subfamily: Pooideae).

#### Description

Lower leaf epidermis composed of long cells, short cells, and stomatal complexes. Long cells narrowly oblong, 37–127 µm long (X_n_ = 78 µm, n = 20), 7–18 µm wide (X_n_ = 13 µm, n = 20), with wavy and thick walls, regularly or irregularly interspersed with short cells, non-papillate. Short cells present, paired. Stomatal complexes 19–39 µm long (X_n_ = 33 µm, n = 20), 9–25 µm wide (X_n_ = 20 µm, n = 20), subsidiary cells usually low dome-shaped or parallel-sided. Macro-hairs absent, micro-hairs present occasionally.

#### Identification

Unearthed epidermis of wheat (*Triticum* sp.) was identified by comparison with illustrations in Chen *et al.* (1993) [30], and Cai & Guo (1995) [32], and epidermis from modern wheat (*Triticum aestivum*) (specimen no. 01941718). The thick cell-walls with constant width, remarkable and large stomatal complexes are characteristic features of *Triticum* epidermis (Fig. 6).

2. *Agropyron mongolicum* (Family: Poaceae, subfamily: Pooideae).

#### Description

Lower leaf epidermis composed of long cells, short cells with pitted-cells, and stomatal complexes. Long cells narrowly oblong, 34–85 µm long (X_n_ = 62 µm, n = 10), 8–25 µm wide (X_n_ = 16 µm, n = 10), with wavy walls, irregularly interspersed with short cells, non-papillate. Short cells present, solitary or occasionally paired, pitted-cells distinctive with round shape having a diameter of 10–24 µm (X_n_ = 17 µm, n = 10). Stomatal complexes 18–32 µm long (X_n_ = 28 µm, n = 10), 15–27 µm wide (X_n_ = 23 µm, n = 10), subsidiary cells usually low dome-shaped or parallel-sided. Macro-hairs absent, micro-hairs present occasionally.

#### Identification

Unearthed epidermis of *Agropyron mongolicum* was identified by comparison with illustrations in Chen *et al.* (1993) [30] and Xie & Yang (1994) [31], and an epidermis from a modern plant (specimen no. 01821117). There are five species in the genus *Agropyron, Agropyron mongolicum* is characterized by pitted-cells, as well as paired short cells present occasionally (Fig. 6).

3. *Phragmites* sp. (Family: Poaceae, subfamily: Arundinoideae).

#### Description

Lower epidermis composed of long cells, short cells, stomatal complexes, and prickle-hairs. Long cells narrowly oblong with coarse wavy walls, non-papillate, 51–97 µm long (X_n_ = 61 µm, n = 20), 5–12 µm wide (X_n_ = 8 µm, n = 20), irregularly interspersed with short cells. Short cells single or paired, silica short cells usually saddle-shaped. Stomatal complexes 12–18 µm long (X_n_ = 15 µm, n = 20), 3–8 µm wide (X_n_ = 5 µm, n = 20), subsidiary cells usually parallel-sided, occasionally low dome-shaped. Prickle-hairs and micro-hairs present occasionally.

#### Identification

Unearthed epidermis of reed (*Phragmites* sp.) was identified by comparison with illustrations in Chen *et al.* (1993) [30], Cai & Guo (1995) [32] and Lin (2008) [42], and an epidermis from modern reed (*Phragmites australis*) (specimen no. 0595279). Arundinoideae are distinct from other subfamilies of the Poaceae in having micro-hairs, no papillae, and saddle-shaped silica cells. *Phragmites* is characterized by the coarse wavy wall of the lower epidermis (Fig. 7).

As a whole, the pollen assemblage of Tuyoq Grottoes is dominated by herbs (70.11%), whereas shrubs and trees are only represented by 15.20% and 7.54% respectively. Apart from widespread Poaceae and *Artemisia*, almost all palynotaxa with the greatest relative abundance are herbs and shrubs mainly found in arid or semiarid areas of the temperate zones: *Artemisia*, Chenopodiaceae, *Ephedra*, *Alhagi*. The leaf epidermis of *Agropyron mongolicum* also indicates arid steppe or sandy land. The few trees such as Moraceae, *Ulmus* and *Populus* in the palynoassemblage indicate the local presence of some deciduous broad-leaved plants. The coniferous pollen of *Pinus* and *Picea* was probably transported to the Turpan Basin by wind [43]. Based on the occurrence of aquatic plants (2.26%) such as *Typha* and Potamogetonaceae, swamp plants (4.00%) as *Phragmites australis* and the presence of Polypodiaceae (0.43%), the Tuyoq Valley is suggested to have represented a moist microenvironment with flowing water while the Turpan Basin was generally arid in the Fifth Century. At the same time, wheat and grape were cultivated in this region as pollen of *Vitis* and leaf epidermis of *Triticum* were found.

### Ancient Vegetation

The A/C ratio (A: *Artemisia*, C: Chenopodiaceae) has been widely used in distinguishing different vegetation of arid areas [44]–[47]. Based on Luo *et al.*’s (2007) [48] study on surface soil pollen of Xinjiang, the A/C ratio (median value) of coniferous forest was 3.47, steppes 1.26, desert 0.57, thus an A/C ratio of 1.8 at the Tuyoq Grottoes is indicative of steppe. Furthermore, *Artemisia*, *Ephedra* and Chenopodiaceae are the most widespread pollen taxa throughout unforested areas of Xinjiang. When these taxa occurred with Asteraceae, Poaceae, Cyperaceae and Brassicaceae as the dominant pollen group, they suggest a meadow-steppe vegetation, whereas an alpine-steppe is indicated when they were found alongside Caryophyllaceae, Rosaceae, Ranunculaceae and Apiaceae [49]. Clearly, the dominant pollen group of Tuyoq Grottoes (Poaceae *Artemisia*, *Ephedra*, Chenopodiaceae, *Ephedra* and *Alhagi*) points to a temperate vegetation of meadow-steppe (Table 3).

**Table 3:** Pollen assemblage characteristics of different modern vegetations in Xinjiang with comparison to Tuyoq Grottoes.

Vegetation characteristics	Forest	Steppe		Desert	Tuyoq Grottoes
A/C ratio*	3.47	1.26		0.57	1.8
Vegetation subtype		meadow-steppe	alpine-steppe		
Dominant pollen group	*Picea*	A,C,E*	A,C,E*	A,C,E*	A,C,E*
	*Larix*	Asteraceae	Caryophyllaceae	*Nitraria*	Asteraceae
	*Betula*	Poaceae	Rosaceae		Poaceae
	*Populus*	Cyperaceae	Ranunculaceae	*Lycium*	*Alhagi*
		Brassicaceae	Apiaceae		

* A: *Artemisia*, C: Chenopodiaceae, E: *Ephedra* [48], [49].

### Ancient Climate

**Figure 8:**
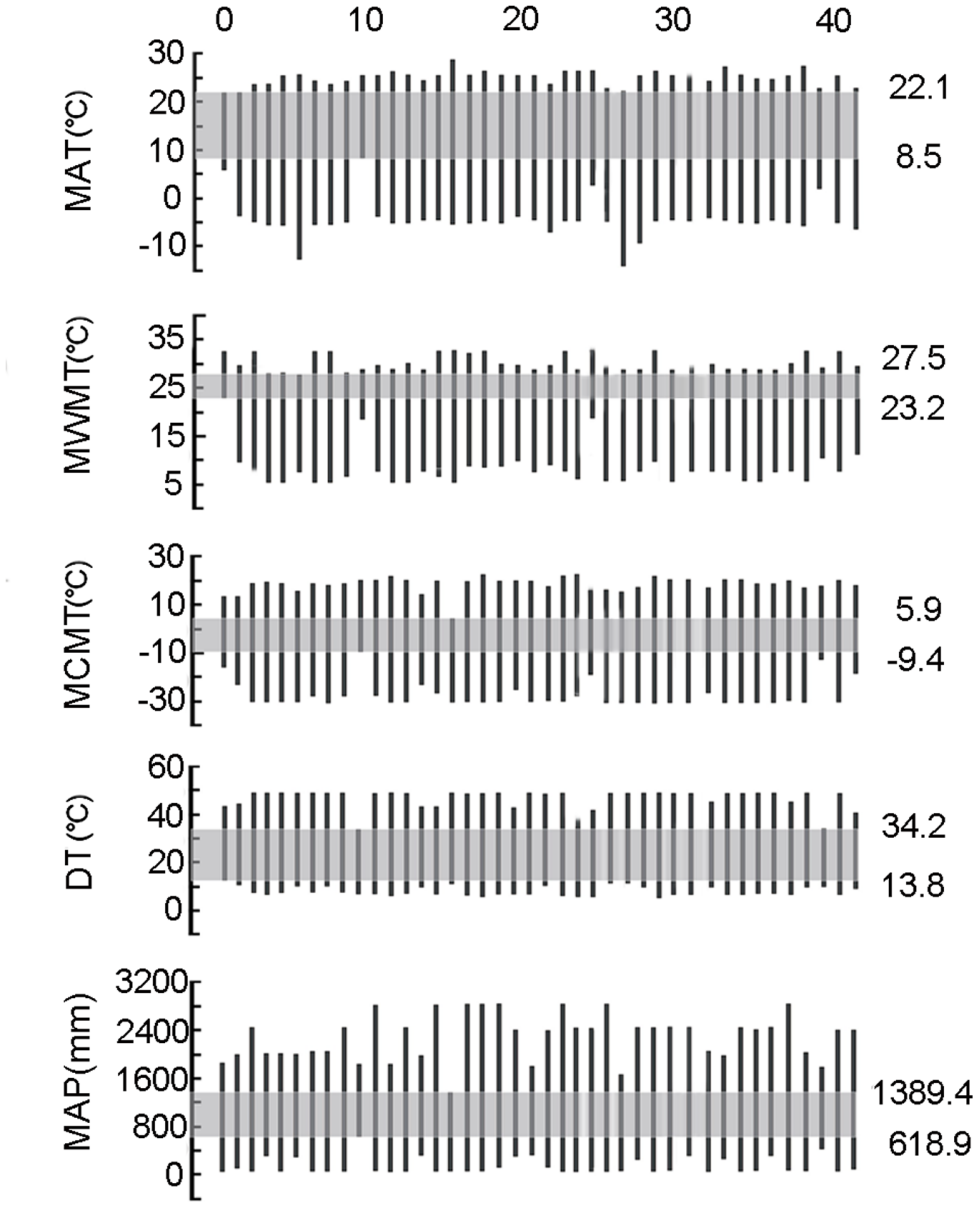
Coexistence intervals of climatic parameters of Tuyoq Grottoes palynoflora. 1. *Alhagi* 2. *Alisma* 3. Apiaceae 4. *Artemisia* 5. Asteraceae 6. *Betula* 7. Boraginaceae 8. Brassicaceae 9. Caryophyllaceae 10. *Castanea* 11. Celastraceae 12. Chenopodiaceae 13. Convolvulaceae 14. *Corylus* 15. Elaeagnaceae 16. *Ephedra* 17. Euphorbiaceae 18. Fabaceae 19. Fagaceae 20. Juglandaceae 21. Lamiaceae 22. Liliaceae 23. Malvaceae 24. *Medicago* 25. Moraceae 26. *Phragmites australis* 27. *Picea* 28. *Pinus* 29. Plantaginaceae 30. Poaceae 31. *Polygonum* 32. Potamogetonaceae 33. *Quercus* 34. Ranunculaceae 35. Rosaceae 36. Rubiaceae 37. Rutaceae 38. *Salix* 39. *Typha* 40. *Ulmus* 41. *Vitis.*

Based on the 41 identified palynomorphs of spermatophytes, five ancient climatic parameters of the Tuyoq Grottoes were reconstructed by CA as follows: MAT = 8.5–22.1°C (15.3°C median value), delimited by *Castanea* and *Alhagi*; MWMT = 23.2–27.5°C (25.4°C), delimited by *Alhagi* and *Betula*; MCMT = −9.4–5.9°C (−1.8°C), delimited by *Castanea* and *Ephedra*; DT = 13.8–34.2°C (24°C), delimited by *Alhagi* and *Castanea*; MAP = 618.9–1389.4 mm (1004 mm), delimited by *Castanea* and *Ephedra* (Fig. 8). Compared with the current meteorologic data (Table 4), the median value of the MAP was much higher 1500 years ago, the MAT, MCMT were respectively 1.4°C and 7.7°C higher 1500 years ago, whereas MWMT and DT were lower than today by 7.3°C and 18.2°C, which indicates that in the Tuyoq region the climate was warmer and wetter with weaker seasonality during the Fifth Century.

**Table 4:** The comparison between the median values of five climatic parameters in Fifth Century Tuyoq Grottoes and the current meteorological data.

Climatic parameters	5^th^ Century	Modern*
MAT(°C)	15.3	13.9
MWMT(°C)	25.4	32.7
MCMT(°C)	−1.8	−9.5
DT(°C)	24.0	42.2
MAP(mm)	1004.2	16.4

* Land climatic data of Turpan Meteorological Station (N 42°56′,E 89°12′), about 47 km east of the Tuyoq Grottoes.

### 3D Reconstruction of Ancient Environment

**Figure 9:**
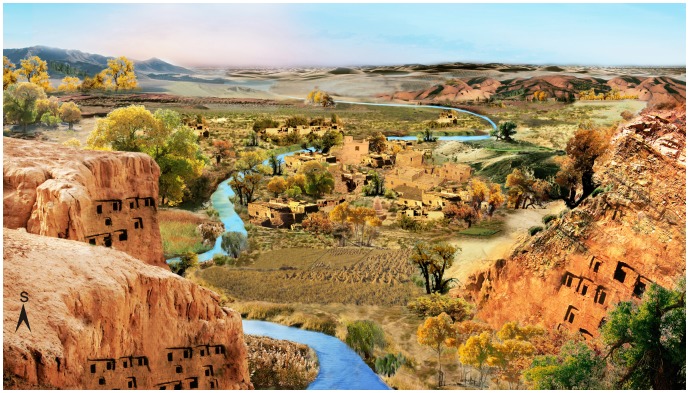
3D reconstruction of environmental landscape of Tuyoq region in the Fifth Century. In this picture, the viewpoint is from north to south.

The environment of Turpan in the Fifth Century was reconstructed and visualized in a 3D presentation based on the investigation of ancient vegetation and climate at the Tuyoq Grottoes (Fig. 9). In the Fifth Century, the landscape of the Tuyoq region was composed of valley, village, mountains and distant desert. Temperate vegetation of meadow-steppe predominated in this area with widespread herbs of *Artemisia*, Chenopodiaceae and numerous shrubs of *Ephedra* and *Alhagi*. The Tuyoq Grottoes were constructed in the cliffs on the sides of the valley. A river flowed through the valley, the village and on to the distant desert. *Phragmites australis*, *Typha* and other aquatic plants grew luxuriantly in the Tuyoq Valley. People lived in the basin oasis nourished by the river, wheat fields and vineyards around the village, along with a few deciduous trees such as *Ulmus*, *Betula*, Moraceae, *Salix* and *Populus*. In the distance, the river is seen vanishing into the desert, while the conifers *Picea* and *Pinus* lived in the surrounding mountains.

## Discussion

### Ancient Pollen of Reed (*Phragmites australis*) Identified by SEM and its Archeological Significance

**Figure 10:**
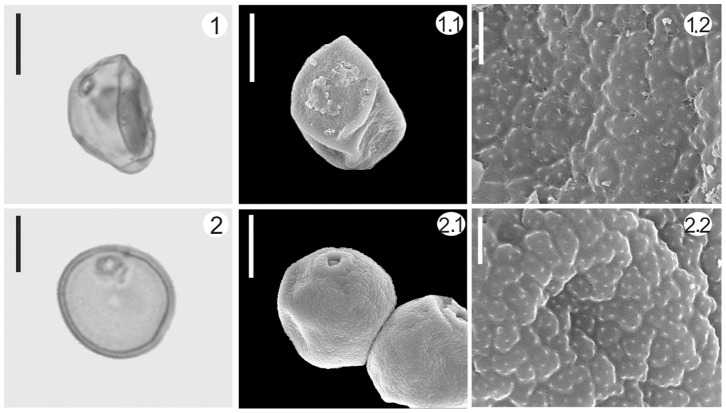
Comparison of ancient reed (*Phragmites australis*) pollen and modern reed pollen. 1. Unearthed pollen of reed (*Phragmites australis*) from Tuyoq Grottoes, 2. Pollen of modern reed (*Phragmites australis*); Scale bar in light microscope (LM) and scanning electron microscopic (SEM) overview = 10 µm, in SEM close-up = 1 µm.

Poaceae pollen have never been used as a diagnostic trait for distinguishing between genera or species since they share the same characteristics (stenopalynous) under light microscopy [50]. In recent years, the scanning electron microscope (SEM) has been increasingly used in pollen identification of cereals and other grasses [51]–[55]. In the Tuyoq Grottoes pollen assemblage, the content of Poaceae pollen reached 26.11%. In order to improve the accuracy of pollen identification, pollen of nine common Poaceae species from the modern Tuyoq region were observed under a SEM, namely *Agropyron cristatum*, *Agropyron mongolicum*, *Festuca arundinacea* subsp. *orientalis*, *Festuca ovina*, *Sorghum bicolor*, *Stipa sareptana*, *Triticum aestivum*, *Zea mays*. In the sample from 1500 years ago, pollen which has the same characteristics as modern reed (*Phragmites australis*) was identified (Fig. 10). Although the identification of a wide range of grasses by their pollen remains a problem, our results are a useful reminder that there is a very real possibility of determining fossil or ancient grass palynotaxa under SEM observation when only a limited number of species is involved. This is promising and practicable for a higher resolution of crop pollen in archaeological research.

### MAP and Water Supplied by Glacial Melt and Underground Water

We noticed that the reconstructed MAP of the Fifth Century was apparently more than sixty times higher than the current precipitation. Actually, in the arid Turpan Basin, most water is supplied by glacial melt from the Tian and Bogeda Mountains and underground water at present. We suppose that the actual rainfall in the Fifth Century could have either been higher than or similar to that of today. If it was similar to today, the reconstructed 1000 mm MAP may not only have consisted of the actual rainfall, but also incorporated glacial melt or underground water, since plants can utilize any source of available water. Although the meteorological precipitation 1500 years ago is hard to evaluate, it is clear that the Tuyoq region had an abundant water supply as a river flowed in the Tuyoq Valley, which was crucial for the oasis development. At the same time, it indicates that a water source such as glacial melt and underground water have to be taken into account when the ancient MAP of an arid area such as Xinjiang is reconstructed using CA.

### Oasis and Buddhism Nourished by Amicable Environment in Turpan

According to the epidermis analysis (Table 2), wheat (*Triticum* sp.) and reed (*Phragmites* sp.) were the most used materials mixed in the clay to strengthen the adobe, whereas *Agropyron mongolicum* and other plants were probably weeds for only a few of them were found. During the early period of the Subeixi Culture (around 700 BC), wheat (*Triticum aestivum*) was a supplemental crop of oases in Turpan [13]. Until the Fifth Century, the frequent occurrence of wheat (*Triticum* sp.) in the Tuyoq Grottoes suggests that it was cultivated on a certain scale,and it became one of the major crops during the Gaochang Period [56]. The development of wheat cultivation and the discovery of grape (*Vitis* sp.) pollen witnessed the blooming of oasis agriculture in this period.

**Figure 11:**
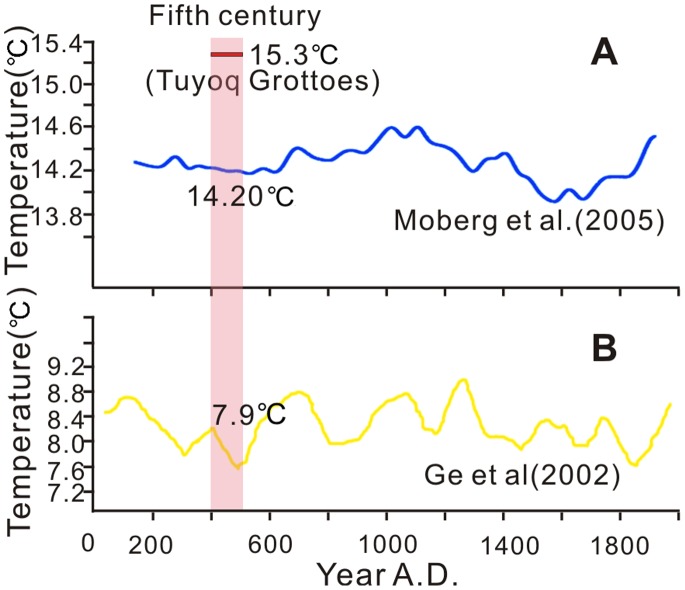
Temperature comparison of the Tuyoq Grottoes and the northern hemisphere (China) in the Fifth Century. (A) Temperature reconstruction of Northern Hemisphere from 133 to 1925 A.D., the mean temperature of fifth century (401–500 A.D.) was 14.20°C [60], (B) Winter half-year-mean temperature reconstruction of eastern China during the past 2000 years, the mean temperature of Fifth Century was 7.9°C [61].

In the Fifth Century, the Xinjiang area experienced a warming climatic tendency [57], [58] whereas the global temperature was undergoing a cooling event around 1.4k BP [59], [60]. The reconstructed MAT of Turpan 15.3°C was 1.1°C warmer than that of the northern hemisphere [60] and 7.4°C warmer than the winter half-year-mean temperature of eastern China [61] in the Fifth Century (Fig. 11), as well as 1.4°C warmer than the current temperature of Turpan 13.9°C [3]. Li (1985) believed that the MAT of the whole of Xinjiang in the Fifth Century was 1–2°C warmer than today [57]; our result is consistent with this conclusion. Since glacial melt was the main water source for the Turpan Basin [4], the alteration of water resources caused by climatic change was the crucial factor for oases development [62]. In the Fifth Century, the warmer climate at the Tuyoq Grottoes supplied abundant glacial meltwater to the valley, which enabled the oasis agriculture to flourish.

The Tuyoq Grottoes, included 94 caves along half a kilometer of cliff face in the Tuyoq Valley, were initiated during the Fifth Century. Construction continued until the Seventh Century [63]. If the oases were not well enough developed to furnish financial and material support, such large scale Buddhist grotto construction would have been impossible.

**Figure 12:**
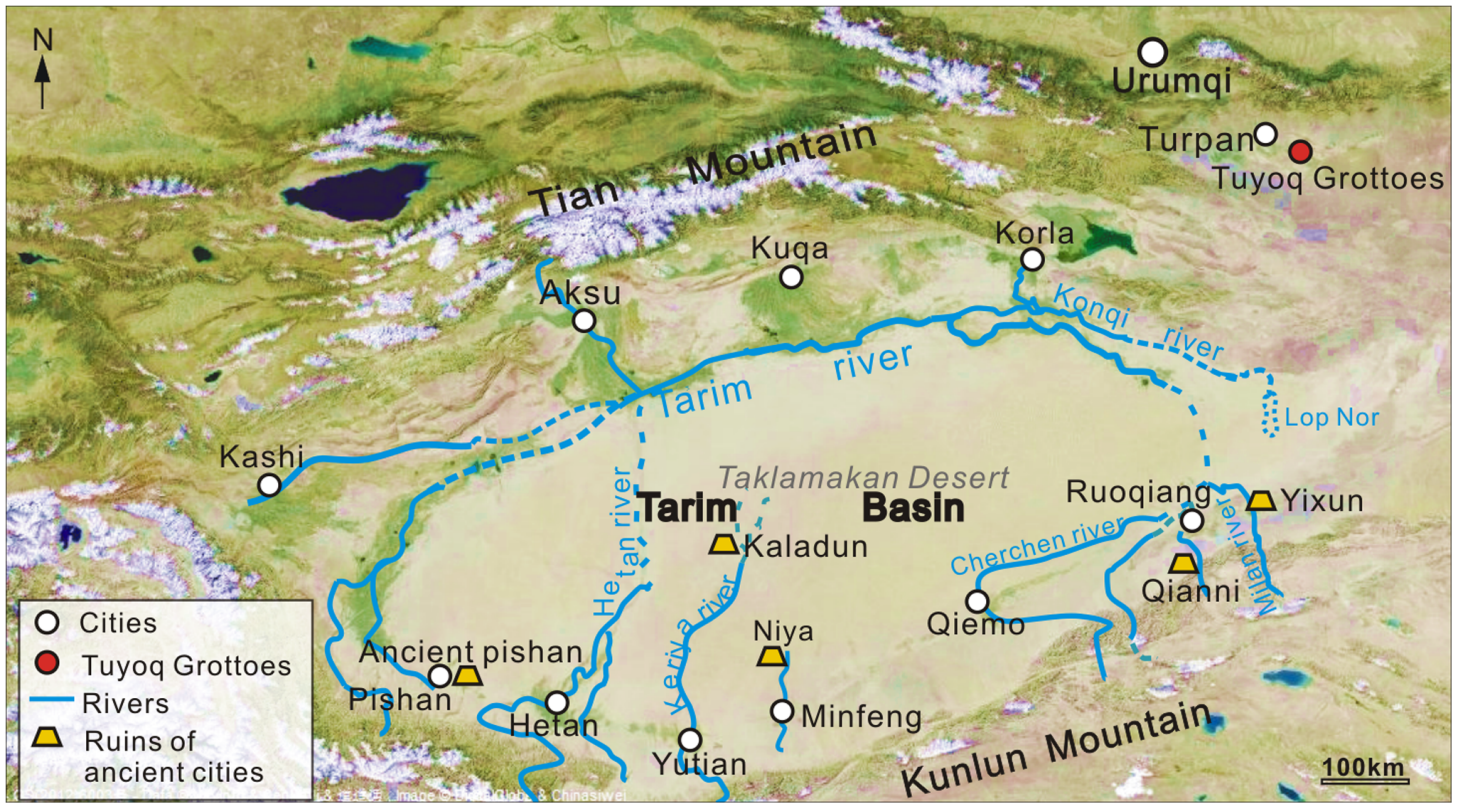
Distribution of ancient cities in southern Tarim Basin abandoned around the Fifth Century (revised from http://map.baidu.com/).

However, in the same period with a similar warm climate in the Taklamakan Desert [58], [64], Niya and a series of ancient cities with Buddhist temples were abandoned (Fig. 12, Table 5). Possible reasons for the abandonment may include: 1) River diversion [65], [66]. As glacial melt increased, increased flow of rivers in the desert basin was prone to cause river diversion but not in an enclosed mountain valley like Tuyoq. 2) River desiccation. When rivers flow across a desert, any increase in temperature would have strengthened evaporation, causing the lower part of the river to dry up. This is considered a possible reason for Niya abandonment [64], [65], [67]. 3) Desertification caused by an expansion of the Taklimakan Desert could explain the abandonment of Kaladun city [66]. 4) War or plague. Based on archaeological observations, we speculate that the abandonment of Niya was probably associated with a war threat or plague, since the unearthed Kharoshthi tablets repeatedly mentioned invasions by the Supis (probably Sump Kingdom of northern Tibet).

**Table 5:** Ancient cities of South Tarim Basin abandoned around the Fifth Century.

Ancient city	Geography	Time of abandonment	Probable reason	Reference
Niya	Lower part of Niya River	4^th^ to 5^th^ century A.D.	River drying up; War or plague (in present work)	[64], [65], [67]
Kaladun	Lower part of Keriya River	4^th^ to 5^th^ century A.D.	River diversion	[65], [66]
Yixun	Middle part of Milan River	5^th^ century A.D.	War	[65]
Ancient Pishan	Lower part of Pishan River	4^th^ to 5^th^ century A.D.	River drying up	[64]
Qianni	Middle part of Ruoqiang River	5^th^ century A.D.	River drying up	[64]
